# Influence of Ellagic Acid and Ebselen on Sperm and Oxidative Stress Parameters during Liquid Preservation of Ram Semen

**DOI:** 10.22074/cellj.2019.5593

**Published:** 2018-11-18

**Authors:** Mustafa Numan Bucak, Mustafa Bodu, Nuri Başpınar, Şükrü Güngör, Pınar İli, Begimay Acibaeva, Tohid Rezaei Topraggaleh, Şükrü Dursun

**Affiliations:** 1 Department of Reproduction and Artificial Insemination, Faculty of Veterinary Medicine, Selcuk University, Konya, Turkey; 2 Department of Biochemistry, Faculty of Veterinary Medicine, Selcuk University, Konya, Turkey; 3 Department of Reproduction and Artificial Insemination, Faculty of Veterinary Medicine, Mehmet Akif Ersoy University, Burdur, Turkey; 4 Pamukkale University, Denizli Health Services Vocational High School, Denizli, Turkey; 5 Department of Embryology, Reproductive Biomedicine Research Center, Royan Inistitute for Reproductive Biomedicine, ACECR, Tehran, Iran; 6 Department of Gynecology and Obstetrics, Faculty of Veterinary Medicine, Aksaray University, Aksaray, Turkey

**Keywords:** Ebselen, Ellagic Acid, Preservation, Ram, Sperm Parameters

## Abstract

**Objective:**

The purpose of the present study was to assess the effects of ellagic acid and ebselen on sperm and oxidative stress parameters during liquid preservation of ram semen.

**Materials and Methods:**

In this experimental study, sixty ejaculates from six mature Merino rams were used. In experiment 1, the ejaculates were diluted in base extender contained ellagic acid at 0 (control), 0.5, 1, and 2 mM. In experiment 2, ebselen at 0 (control), 10, 20, and 40 μM were added to the extender. Sperm motility, viability, mitochondrial membrane potential, DNA integrity, lipid peroxidation (LPO), the antioxidant potential (AOP), and total glutathione (tGSH) were evaluated at 0, 24, 48, and 72 hours of preservation.

**Results:**

Supplementation of ellagic acid at 1 and 2 mM resulted in higher sperm motility and viability at 0 hours of storage. Ellagic acid at 2 mM led to higher motility and viability compared to controls after 0, 24, and 48 hours of preservation and increased AOP after 24 and 72 hours. Higher tGSH was at 1 mM ellagic acid, compared to control after 72 hours. Addition of ebselen at a concentration of 40 μM increased motility at 24 and 48 hours and 10 μM produced the same effect after 48 and 72 hours of storage as well as higher viability, compared to the controls after 0 hours of storage. Sperm DNA integrity was significantly improved after 24, 48, and 72 hours with the addition of ebselen at 10 μM, and after 72 hours at 40 μM. Addition of 40 mM ebselen also reduced the LPO levels after 24 hours of storage compared to the controls.

**Conclusion:**

The results showed that supplementation of ellagic acid and ebselen in semen extender has a potential effect on sperm and oxidative stress parameters during liquid preservation of ram semen.

## Introduction

Artificial insemination is a valuable tool that plays a critical role in the reproduction of small ruminants. It facilitates the distribution of semen from superior sires to a large number of females, allowing the improvement of desirable characteristics, e.g. milk, meat, and wool production (1, 2). Artificial insemination, which is done using fresh, liquid preserved or frozen-thawed semen, improves the lambing rate in sheep breeding (1, 3). Successful liquid preservation of ram semen is achieved by providing the necessary environmental conditions. These conditions include: i. The development of extenders that keep functional sperm parameters, ii. Minimizing the generation of reactive oxygen species (ROS), and the prevention of oxidative stress (1, 4). 

Ram sperm is more susceptible to ROS than the other species due to a higher ratio of polyunsaturated/saturated fatty acids and a lower cholesterol/phospholipid molar ratio (5). The polyunsaturated fatty acids (PUFAs) render the sperm membrane a high vulnerability to the sperm membrane to be attacked by ROS resulting in functional impairment of the sperm cells (6). The effect of ROS generated during the peroxidation of sperm membrane lipids leads to poor quality semen, decreased motility, damaged DNA, disrupted acrosome reaction, and capacitation (7). Normally, the semen contains antioxidants, including taurine, catalase, glutathione, glutathione peroxidase (GPx), and superoxide dismutase that can oppress the lipid peroxidation (LPO) and excessive ROS generation (5). However, this endogenous antioxidative capacity may be insufficient in preventing the LPO during a prolonged storage or an unfrozen state (8). Reports have indicated that the addition of antioxidants, such as methionine, dithioerythritol, taurine, lipoic acid, lycopene, cysteamine, and reduced glutathione into the sperm extenders decreased the impact of different oxidants and protected the sperm cells from oxidative damage during liquid preservation of ram spermatozoa (9-12). 

Ellagic acid is a natural phenol compound with a polyphenolic structure and a strong antioxidant. The cryoprotective and antioxidative properties of ellagic acid have been previously reported in a reduction of the LPO and increment of the total glutathione (tGSH) and GPx levels in rats (13). Also, oral administration of ellagic acid has been observed to increase epididymal sperm motility and its concentration in rats (14). Ellagic acid has also demonstrated the protective effects against adriamycin, which has the disrupting effects on epididymal sperm quality parameters and the LPO in the rat testis (15). 

Ebselen (2-phenyl-1,^2^-benzisoselenazol-3[2H]-1) is a seleno-organic molecule which scavenges ROS by mimicking the GPx activity (16). Ebselen, with its cyto/ neuroprotective effects, has been observed to reduce the DNA damage and oxidative stress caused by hydrogen peroxide generated in hamster lung fibroblasts (17). Also, ebselen was reported to reduce the LPO levels, demonstrating the protective effects in the murine cardiovascular system (18). In humans, ebselen has been reported to be a substrate for the thioreductase system and a mimetic for the GPx activity in the presence of glutathione and glutathione reductase (GR) (16).

We found that there has been no research conducted to compare the influence of the antioxidants, ellagic acid, and ebselen at different doses during liquid preservation of ram sperm. Therefore, the aim of this study is to evaluate the effects of ellagic acid and ebselen at different doses which were added to Tris extender to monitor ram sperm motility, viability, mitochondrial membrane potential, the DNA integrity as well as oxidative stress parameters (total antioxidant potential, lipid peroxidation, and total glutathione) up to 72 hours of liquid storage at 5°C.

## Materials and Methods

All chemicals were obtained from Sigma Aldrich (St. Louis, MO, USA) unless otherwise indicated. In this experimental study, employed protocols were approved by the Animal Ethics Committee of the Veterinary faculty of Selcuk University, Turkey.

### Animals and semen collection 

The study was conducted at the Bahri Dagdas International Agricultural Research Institute (Konya, Turkey) during the breeding season (autumn to early winter) 2016. A total number of 60 ejaculates from six mature Merino rams (2 and 3 years of age) were collected twice a week using an artificial vagina. Ejaculates meeting the following criteria were evaluated: a volume of 0.52 mL, a minimum sperm concentration of 2×10^9^ sperm/ mL, and motility of >80 %. Semen samples of six rams were pooled and ten pooled samples were used in each experiment. 

### Semen processing and experimental design

Semen volume was measured with a graduated conical tube and sperm concentration was determined using a hemocytometer. A Tris-based extender (Tris 297.58 mM, citric acid 96.32 mM, fructose 86.66 mM, egg yolk 15 % (v/v) at pH=6.8) was used as a base extender. This study included two experiments carried out in succession. 

Experiment 1: Each pooled ejaculate was diluted in a Tris-based extender (37°C) and divided into four equal experimental groups contained ellagic acid at 0 (control), 0.5, 1, and 2 mM with a final sperm concentration of approximately 400×10^6^ cells/mL. 

Experiment 2: Ebselen at 0 (control), 10, 20 and, 40 µM was used as an additive to the extender and the above procedure was applied for dividing and extending the semen.

For both of experiments, diluted semen samples with antioxidants were kept in 15-mL plastic tubes and cooled down from 37 to 5°C (within one hour) and kept at 5°C during liquid preservation for up to 72 hours. The sperm quality and oxidative stress parameters were determined after 0, 24, 48, and 72 hours of liquid storage in both experiments. The procedure was repeated 10 times for each experiment.

### Semen evaluation 

#### Evaluation of sperm parameters during liquid preservation of ram semen

##### Motility evaluation 

Sperm motility was measured using a phase-contrast microscope (×200 magnification). Five microliters of sample were dropped onto a pre-warmed microscope slide and then covered with a coverslip. For each semen sample, sperm motility was measured in three different microscopic fields. The mean of the three successive estimations was recorded as a final motility score (19). 

#### Assessment of sperm plasma membrane integrity (viability) 

Sperm plasma membrane integrity was assessed using a Sperm Viability Kit (SYBR- 14/PI Molecular Probe: L 7011 Invitrogen, Carlsbad, CA) following a modified protocol from Garner et al. (20). A working solution of SYBR-14 was diluted at a ratio of 1:10 with dimethyl sulfoxide (DMSO) (Applichem A3006) and propidium iodide (PI) was dissolved in distilled water at 2 mg/mL. The semen sample was diluted at 1:3 with Tris stock solution (Tris 297.58 mM, citric acid 96.32 mM, fructose 86.66 mM) and then 30 µL of the diluted semen was mixed with 6 µL of SYBR-14 and 2.5 µL of PI. The sample was mixed and incubated at 37°C in the dark place for 20 minutes. Then, 10 µL of Hancock solution was prepared according to the protocol from Schäfer et al in order to stop the sperm motion (21). A drop of 2.5µL sample was placed on a microscope slide and covered with a coverslip. At least 200 spermatozoa were examined at 1000x magnification under a fluorescence microscope (Leica DM 3000 Microsystems GmbH, Ernst-Leitz- Straße, Wetzlar, Germany; excitation at 450-490 nm, emission at 520 nm) to evaluate the sperm membrane integrity. The sperms exhibiting green-red or red color were considered membrane damaged (not viable), while those displaying green color were considered intact membranes spermatozoa (viable) (22).

### Evaluation of sperm mitochondrial activity

For the assessment of sperm mitochondrial activity, a stock solution contained 5, 5', 6, 6'-Tetrachloro-1, 1', 3, 3' tetraethyl-benzimidazolylcarbocyanine iodide (1.53 mM) (T3168 JC-1, Invitrogen, Carlsbad, CA) in dimethyl sulfoxide (DMSO) was prepared. The semen sample was diluted 1:3 with Tris stock solution. Subsequently, 2.5 µL of JC-1 and 2.5 µL PI were added to 300 µL of diluted samples and gently mixed and incubated at 37°C for 20 minutes in the dark place. Then, 10 µLof Hancock solution was added to stop sperm motion. A drop of (2.5 µL) the sample was placed on a microscope slide and covered with a coverslip. At least 200 sperm cells were observed at ×1000 magnification under a fluorescence microscope (Leica DM 3000 Microsystems GmbH, Ernst-Leitz- Straße, Wetzlar, Germany; excitation at 450-490 nm, emission at 520 nm) to assess the mitochondrial activity. A high level of yellow/orange and green fluorescence in sperm midpiece indicated the high and low mitochondrial activity, respectively (12).

### Evaluation of sperm DNA damage 

Single cell gel electrophoresis (COMET) assay was used for the evaluation of sperm DNA damage. The semen samples were centrifuged at 600x g for 10 minutes at 4°C and the remaining pellets were resuspended in phosphate buffered saline (PBS). Pre-cleaned slides were coated with a layer of 1% solution normal melting agarose in PBS and dried at room temperature. Eighteen microliters of the sperm/PBS mixture were mixed with a 0.75% solution of low melting agarose (50 µL) and placed onto first agarose layer (approximately 1×10^5^ cells). The slides were kept at 4°C for 20 minutes. Then coverslips were removed and slides were immersed in lysis buffer (2.5 M NaCl, 10 mM Tris, 100 mM Na_2_.EDTA, 10 mM Trizma base, 1% N-lauroyl sarcosine, 1% Triton X-100, 70 mM DL Dithiothreitol, pH=10.0) contained 20 µg/mL proteinase K (Vivantis) for 2 hours at 37°C. Then slides were horizontally placed in electrophoresis buffer [1X Tris/Borate/EDTA (TBE) buffer, pH=8] and electrophoresis was performed at room temperature at 25 Volts for 20 minutes. Following the electrophoresis, slides were air-dried and stained with 50 µL of 8 µg/mL ethidium bromide and covered with a coverslip. 

The images of 200 randomly selected sperm nuclei were evaluated through a visual observation (×1000 magnification) using a fluorescent microscope (Leica DM 3000 Microsystems GmbH, Ernst-Leitz-Straße, Wetzlar, Germany). Each image was classified as damaged (sperm showing a “comet” pattern possessed a tail of fragmented DNA migrated from the sperm head) and undamaged (whole sperm heads without a comet tail). All data were expressed as the mean percentages of the undamaged sperm heads ± SEM (22). 

### Evaluation of oxidative stress parameters during liquid preservation of ram semen

Diluted semen samples were centrifuged at 800 xg for 10 minutes at 4°C and the spermatozoa were washed twice with saline through the procedure mentioned above. Then, the supernatant was removed, and pellets were resuspended in 500 µL of PBS, transferred into a 2-mL beaker on ice water and sonicated with a probe (Bandelin Sonopuls, Bandelin Electronic HeinrichstraBe, D-12207, Gerate-Typ: UW 2070, Pro-Nr. 51900037369.004, Berlin) for 10 seconds on ice. This procedure was repeated 5 times at intervals of 30 seconds. 10 µL of Butilated Hydroxi Toluen (BHT, B-1378) was added to 120 µL of sonicated homogenate to avoid further oxidation and stored at -86°C until the LPO assay. The remaining sonicated homogenate was centrifuged at 8000 xg for 5 minutes at 4°C. The supernatant was collected and stored at -86°C until AOP and tGSH assays (22).

### Determination of AOP, LPO, and tGSH levels

The AOP, LPO, and tGSH levels were measured with the use of the AOP-490^TM^, LPO-586^TM^ and GSH-420^TM^ kits respectively (Oxis ResearchTM, Bioxytech, CA, 92202, USA) according to manufacturer’s instructions. Absorption was measured at 490 nm, 586 nm and 405 nm for AOP, LPO, and tGSH, respectively using a spectrophotometer (UV 2100 UV-VIS Recording Spectrophotometer Shimadzu, Japan). The results of the AOP assay were expressed as mmol (10^9^ cells/mL), LPO and tGSH were expressed as µmol (10^9^ cells/mL) (22).

### Statistical analysis

The test was repeated 10 times for each experiment. The results were expressed as the mean ± SEM. The means were analyzed by analysis of variance (ANOVA) followed by Duncan’s post hoc test to determine the significance in all the parameters within all groups using the SPSS/PC computer program (Version 15.0, SPSS, Chicago, IL). The differences were considered to be statistically significant when the P value was less than 0.05.

## Results

**Fig.1:**
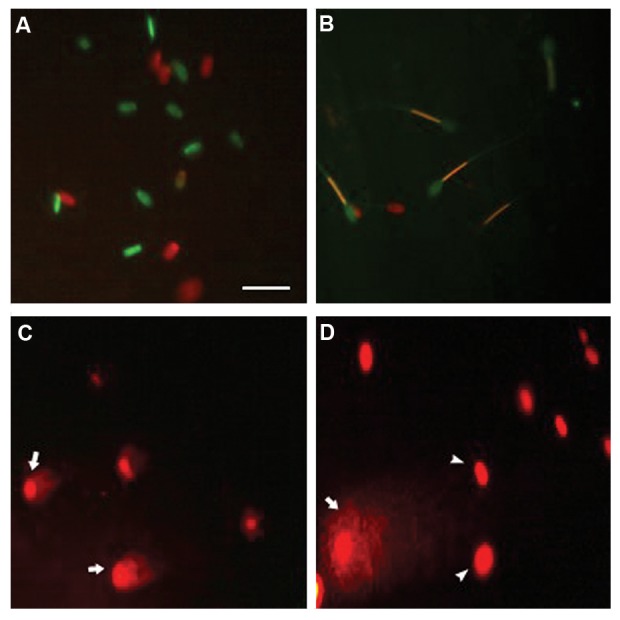
Viability, mitochondrial activity, and DNA integrity of ram spermatozoa. A. Sperm viability assessed by SYBR/PI. Sperm displaying green-red or red was considered as membrane damaged (not viable), while sperm displaying green was considered to be intact membrane (viable), B. JC-1/PI staining for mitochondrial activity. Yellow/orange and green fluorescence associated with midpiece of sperm indicated a high and a low mitochondrial activity, respectively. No fluorescence associated with the midpiece of sperm indicated no mitochondrial activity, C and D. Sperm DNA damage assessed using COMET assay. Sperm heads with undamaged (arrow) DNA and with damaged (arrowhead) DNA (scale bar: 20 µm).

Sperm motility, SYBR/PI, JC-1/PI, and the DNA integrity rate of Merino ram semen supplemented with different concentrations of ellagic acid are shown in Table 1 and oxidative stress parameters in Table 2. The sperm viability, the mitochondrial activity, and the DNAintegrity are shown in Figure 1. The extender supplemented with 1 and 2 mM doses of ellagic acid resulted in a higher motility and the percentage of viable sperm in comparison to the control groups at 0 hours of storage (P<0.05). Ellagic acid at 2 mM led to a higher motility and viability rates when compared to controls during 0, 24, and 48 hours of liquid storage (P<0.05). When ellagic acid was added, the rates of the sperm mitochondrial activity and the DNA integrity were not statistically improved for any of the storage periods at 5°C. Ellagic acid at 2 mM concentration increased the AOP activity at 24 and 72 hours and tGSH after 72 hours at 1 mM, in comparison with the controls (P<0.05). 

**Table 1:** Mean (± SE) sperm motility, SYBR/PI, JC-1/PI and DNA integrity (%) of Merino ram semen supplemented with different concentrations of ellagic acid for different storage times at 5℃


Groups	0 hour				24 hours				48 hours				72 hours			
	Motility	JC-1/PI	SYBR/PI	DNA Integrity	Motility	JC-1/PI	SYBR/PI	DNA Integrity	Motility	JC-1/PI	SYBR/PI	DNA Integrity	Motility	JC-1/PI	SYBR/PI	DNA Integrity

Control	79.1 (± 2.0^a^)	84.0 (± 2.8)	71.3 (± 2.1^a^)	85.5 (± 3.3)	70.8 (± 0.8^a^)	77.8 (± 4.6)	64.1(± 2.6^a^)	70.0 (± 7.0)	63.3 (± 1.6^a^)	72.5 (± 6.9)	54.0 (± 3.8^a^)	67.6 (± 5.4)	54.1 ± 3.9)	56.7 ± 6.2)	58.9 (± 5.0)	67.3 (± 5.8)
0.5 mM	82.5 (± 1.1^ab^)	82.6 (± 4.9)	75.6 (± 2.5^ab^)	81.0 (± 3.6)	73.3 (± 1.6^ab^)	75.6 (± 5.8)	65.9 (± 4.1^ab^)	66.8 (± 5.8)	67.5 (± 1.1^ab^)	70.0 (± 5.1)	58.1 (± 2.6^a^)	66.3 (± 4.6)	61.6 ± 2.1^ab^)	59.3 ± 6.5)	55.3 (± 2.2)	67.8 (± 3.8)
1 mM	85.8 (± 1.5^b^)	85.3 (± 2.9)	78.9 (± 1.6^b^)	82.1 (± 3.0)	73.3 (± 1.0^ab^)	80.6 (± 4.3)	63.1 (± 3.0^ab ^)	73.0 (± 2.4)	65.8 (± 2.0^ab^)	70.9 (± 6.8)	58.4 (± 2.6^a^)	69.0 (± 2.6)	57.5 ± 2.5^ab^)	64.1 ± 7.3)	57.0 (± 4.1)	70.3 (± 3.5)
2 mM	85.0 (± 1.2^b^)	85.2 (± 4.9)	78.7 (± 2.0^b^)	85.0 (± 2.4)	75.0 (± 0^b^)	78.1 (± 5.8)	73.0 (± 1.9^b^)	76.5 (± 2.9)	69.1 (± 2.0^b^)	71.7 (± 5.4)	69.4 (± 2.6^b^)	78.0 (± 3.0)	63.3 ± 1.6^b^)	69.2 ± 6.2)	64.0 (± 1.8)	70.5 (± 2.9)


Means with different letters (a, b) in the same column demonstrate significant differences (P<0.05).

The sperm motility, SYBR/PI, JC-1/PI, and the sperm DNA integrity in Merino ram semen supplemented with different concentrations of ebselen are shown in Table 3 and oxidative stress parameters in Table 4. The extender supplemented with 40 µM ebselen led to higher motility rates in comparison with the control groups at 24 and 48 hours and at 48 and 72 hours time points with 10 µM. Ebselen at a concentration of 10 µM resulted in a higher viability rate compared to control group at 0 hours of storage. The DNA integrity analysis revealed that ebselen provided a better protective effect on the DNA integrity compared to other groups at 10 µM after 24, 48 and 72 hours, and after 72 hours at 40 µM (P<0.05). Regarding the biochemical parameters, only a dose of 40 mM ebselen reduced the LPO levels during 24 hours of liquid storage compared to controls (P<0.05). 

**Table 2:** Mean (± SE) LPO (µmol, 10^9^ Cells/ml), tGSH (µmol, 10^9^ Cells/ml) and AOP (mM×10^9^) levels of Merino ram semen diluted with different doses of ellagic acid at different storage times at 5℃


Groups	0 hour			24 hours			48 hours			72 hours		
	AOP	LPO	tGSH	AOP	LPO	tGSH	AOP	LPO	tGSH	AOP	LPO	tGSH

Control	36.0 (± 5.0)	70.5 (± 32.9)	2142.5 (± 802.7)	28.4 (± 3.1^a^)	57.7 (± 8.6)	1808.4 (± 320.6)	29.89 (± 4.6)	55.0 (± 11.5)	1644.0 (± 184.2)	28.9 (± 4.1^a^)	41.4 (± 5.3)	1024.4 (± 187.2^a^)
0.5 mM	66.5 (± 11.0)	100.8 (± 84.9)	2087.2 (± 1135.0)	58.5 (± 12.4^ab^)	74.7 (± 14.5)	1661.7 (± 276.8)	62.19 (± 12.1)	67.0 (± 14.0)	1445.4 (± 167.0)	63.5 (± 12.5^ab^)	84.1 (± 16.0)	1655.4 (± 240.1^ab^)
1 mM	55.1 (± 10.1)	58.9 (± 19.7)	2483.0 (± 978.5)	82.8 (± 18.8^ab^)	71.3 (± 7.5)	2392.0 (± 479.0)	65.24 (± 10.6)	53.3 (± 10.0)	2515.6 (± 725.0)	65.9 ± 12.9^ab^)	78.9 (± 17.0)	2630.9 (± 615.3^b ^)
2 mM	83.5 (± 18.3)	89.5 (± 31.6)	2494.7 (± 2203.0)	91.4 (± 18.2^b^)	70.6 (± 17.3)	2197.8 (± 588.9)	56.75 (± 12.4)	68.8 (± 12.0)	1402.9 (± 131.3)	71.7 ± 9.7^b^)	64.2 (± 15.2)	1718.2 (± 335.9^ab^)


Means with different letters (a, b) in the same column demonstrate significant differences (P<0.05). LPO; Lipid peroxidation, tGSH; Total glutathione, and AOP; Total antioxidant potential.

**Table 3:** Mean (± SE) sperm motility, SYBR/PI, JC-1/PI and DNA integrity (%) of Merino ram semen supplemented with different concentrations of ebselen for different storage times at 5℃


Groups	0 hour				24 hours				48 hours				72 hours			
	Motility	JC-1/PI	SYBR/PI	DNA Integrity	Motility	JC-1/PI	SYBR/PI	DNA Integrity	Motility	JC-1/PI	SYBR/PI	DNA Integrity	Motility	JC-1/PI	SYBR/PI	DNA Integrity

Control	89.1 (± 0.8)	64.6 (± 2.4)	85.5 (± 1.3^a^)	72.0 (± 1.3)	78.3 (± 1.0^a^)	63.0 (± 1.1)	81.2 (± 1.8)	58.5 (± 3.1^a^)	69.1 (± 0.8^a^)	62.8 (± 1.5)	79.6 (± 2.3)	51.5 (± 1.6^a^)	63.3 ( ± 1.0^a^)	59.8 (± 2.2^ab^)	72.4 (± 1.9)	44.0 (± 2.6^a^)
10 µM	90.0 (± 0)	71.5 (± 1.3)	93.3 (± 1.8^b^)	68.2 (± 1.5)	80.8 (± 0.8^ab^)	64.7 (± 1.7)	83.9 (± 2.1)	65.3 (± 1.1^b^)	73.3 (± 1.0^b^)	62.7 (± 2.2)	80.0 (± 1.1)	58.0 (± 0.4^b^)	69.1 (± 1.0^b^)	63.4 (± 2.7^ab^)	73.0 (± 1.5)	54.3 (± 0.8^b^)
20 µM	90.0 (± 0)	65.5 (± 1.9)	88.3 (± 0.9^ab^)	70.3 (± 2.9)	81.6 (± 1.0^ab^)	60.7 (± 0.6)	81.0 (± 3.3)	63.0 (± 0.8^ab^)	72.5 (± 1.1^ab^)	65.1 (± 1.6)	73.7 (± 2.7)	54.1 (± 1.2^a^)	65.8 (± 1.1^a^)	53.7 (± 1.5^a^)	70.9 (± 3.3)	51.1 (± 1.3^ab^)
40 µM	90.0 (± 0)	67.6 (± 2.6)	88.6 (± 2.7^abc^)	71.5 (± 2.9)	82.6 (± 1.2^b^)	61.2 (± 2.8)	79.3 (± 1.4)	62.6 (± 1.7^ab^)	73.3 (± 1.0^b^)	62.7 (± 1.5)	79.8 (± 1.4)	53.1 (± 1.0^a^)	65.0 (± 1.1^a^)	59.0 (± 0.9^b^)	70.0 (± 2.4)	53.3 (± 4.2^b^)


Means with different letters (a, b) in the same column demonstrate significant differences (P<0.05).

**Table 4:** Mean (± SE) LPO (µmol, 10^9^ Cells/ml), tGSH (µmol, 10^9^ Cells/ml) and AOP (mM×10^9^) levels of Merino ram semen diluted with different doses of ebselen at different storage times at 5℃


Groups	0 hour			24 hours			48 hours			72 hours		
	AOP	LPO	tGSH	AOP	LPO	tGSH	AOP	LPO	tGSH	AOP	LPO	tGSH

Control	43.0 (± 7.2)	114.9 (± 25.3)	8088.6 (± 1784.5)	39.5 (± 4.3)	310.2 (± 52.4^b^)	4860.2 (± 1063.2)	33.9 (± 5.4)	204.3 (± 32.3)	5627.3 (± 1362.1)	29.9 (± 4.2)	101.8 (± 11.9)	5214.2 (± 1376.6)
10 µM	45.2 (± 4.5)	156.8 (± 25.1)	10700.5 (± 4287.1)	38.5 (± 8.7)	186.1 (± 31.2^ab^)	4276.1 (± 952.6)	33.8 (± 5.1)	194.5 (± 41.5)	6468.4 (± 1120.8)	24.3 (± 6.5)	86.5 (± 9.2)	5626.2 (± 854.7)
20 µM	28.9 (± 2.2)	122.3 (± 67.1)	5411.9 (± 989.1)	35.6 (± 3.1)	313.8 (± 39.1^b^)	4947.1 (± 865.5)	24.3 (± 3.8)	153.0 (± 21.3)	4790.2 (± 989.5)	35.7 (± 6.9)	104.9 (± 19.9)	4688.7 (± 1391.9)
40 µM	38.1 (± 2.0)	171.6 (± 45.9)	8220.4 (± 2761.6)	47.7 (± 6.7)	153.0 (± 22.9^a^)	4277.7 (± 605.6)	37.9 (± 6.6)	157.4 (± 17.0)	6198.4 (± 1804.5)	26.1 (± 4.8)	92.1 (± 9.9)	4174.3 (± 727.7)


Means with different letters (a, b) in the same column demonstrate significant differences (P<0.05). AOP; Total antioxidant potential, LPO; Lipid peroxidation, and tGSH; Total glutathione.

## Discussion

Ram spermatozoa are more susceptible to ROS induced damages (decreased membrane integrity, motility, DNA intactness, and consequently low fertility) due to the high amounts of PUFAs in membrane structure (23, 24). Supplementation of appropriate antioxidants in semen extender prior to liquid storage prevents these damages (10, 25). The following study was conducted to find out which antioxidants demonstrate the highest effective protection against the sperm damage during liquid preservation. Our results demonstrate an improvement of sperm motility owing to the addition of ellagic acid to the extender at 1 and 2 mM doses. However, at a concentration of 2 mM higher effectiveness was shown in extending the duration of effective liquid storage.

It is assumed that ellagic acid with its phenolic structure may increase the antioxidative capacity by protecting against the harmful effects of free radicals. Sperm motility is linked to three main factors: regulation, structural integrity, and continuity of the energy. While the flagellar part is responsible for motility, the principal portion of the spermatozoa is in charge of hyperactivation (26, 27). However, PUFAs in the middle portion render this structure a sensitivity to free radical attacks (27). Ellagic acid (at 2 mM dose) is thought to protect this functional structure of the middle part of the spermatozoa, which consists of highly vulnerable PUFAs and improve the sperm motility during liquid storage and increase the total AOP levels during 24 and 72 hours of storage.

These results are in accordance with Türk et al. (14), who reported the effect of ellagic acid on increase in the epididymal sperm motility in rats. In another study, Çeribasi et al. (15) displayed the effects of ellagic acid on the ameliorating adriamycin- induced high LPO levels and apoptosis in rats. Also, Ömür and Coyan reported improving effects of ellagic acid on ram semen after freezing/thawing (28). With all the results obtained from these studies, it can be postulated that ellagic acid may be a powerful antioxidative agent, protecting the cell membranes from cryoinjury. 

A seleno-organic molecule, ebselen has cyto/ neuroprotective effects through reducing the DNA damage and oxidative stress caused by the generation of hydrogen peroxide in hamster lung fibroblasts (17). In the current study, ebselen (40 µM dose) reduced the LPO levels at 24 hours of liquid storage in ram semen. In humans, ebselen has been reported to be a substrate for the thioreductase system and mimics the GPx activity in the presence of GSH and GR (16, 29). The different effects of ebselen may be due to cell metabolism since ebselen increases the LPO levels in human multiple myeloma cells at a dose of 10 mM (30). In the same study, ebselen decreased the mitochondrial activity and induced apoptosis. In the current study, however, ebselen did not affect the mitochondrial activity. As spermatozoa have 2275 mitochondria, we speculated that the different effects of ebselen on the spermatozoa may stem from variations in the metabolism. 

During liquid storage, with the extension of time, the motility, viability, and the DNA integrity rates were decreased. It was observed that ebselen improved the motility and the DNA integrity rates. It can be argued that a positive effect of ebselen on sperm motility is mediated via the retention of the DNA integrity, rather than the reduction of the LPO levels. Furthermore, the LPO may not be a major factor influencing the sperm motility during the cooled storage. This is in contrast to the findings of Baumber et al. (31) who demonstrated a markedly decline in equine sperm motility associated with ROS. This study was contradicted to those that indicate supplementation of boar and canine semen with antioxidants increases the sperm motility through the prevention of ROS generation (32, 33). The different observations in the susceptibility of sperm to oxidative stress may be due to the differences in experimental methodology and animal species. 

## Conclusion

Ellagic acid at 2 mM led to a higher motility and viability rates compared to the controls during 0, 24 and, 48 hours of liquid preservation. Ebselen led to a higher motility rate in comparison to the control groups at 24 and 48 hours at a dose of 40 µM and after 48 and 72 hours at 10 µM. The DNA integrity analysis showed that ebselen provided a protective effect on DNA integrity in comparison to the other groups for 24, 48, and 72 hours at 10 µM, and for 72 hours at 40 µM. Only a dose of 40 µM ebselen reduced the LPO levels during 24 hours of liquid preservation, compared to the control group. Ellagic acid led to increasing in the AOP activity in comparison to the control groups after 24 and 72 hours at 2 mM and increased tGSH after 72 hours at a concentration of 1 mM. Addition of these antioxidants prior to the freezing process is suggested to enhance the sperm liquid storage techniques in the sheep breeding industry. Furthermore, future research should focus on a better understanding of the molecular and biochemical mechanisms of the cryoprotective effects of antioxidants, such as ellagic acid and ebselen in a cooled storage of ram semen. 
